# Synchronous Hepatocellular Carcinoma and Cholangiocellular Carcinoma on ^18^F-FDG PET/CT

**DOI:** 10.4274/mirt.93723

**Published:** 2018-10-09

**Authors:** Ezgi Başak Erdoğan, Hacı Mehmet Türk, Ertuğrul Tekçe, Mehmet Aydın

**Affiliations:** 1 Bezmialem Vakıf University Faculty of Medicine, Department of Nuclear Medicine, İstanbul, Turkey; 2 Bezmialem Vakıf University Faculty of Medicine, Department of Medical Oncology, İstanbul, Turkey; 3 Bezmialem Vakıf University Faculty of Medicine, Department of Radiation Oncology, İstanbul, Turkey

**Keywords:** Synchronous tumors, hepatocellular carcinoma, cholangiocellular carcinoma, ^18^F-FDG PET/CT

## Abstract

A 43-year-old male patient presented with a mass lesion on the right liver lobe, segment 5, in radiological imaging and elevated alpha-fetoprotein levels (323 ng/mL) compatible with hepatocellular carcinoma (HCC). Positron emission tomography/computed tomography (PET/CT) images showed background level ^18^F-FDG uptake in the mass lesion. In addition, a secondary focus of increased ^18^F-FDG uptake was detected on the left liver lobe, segment 2, approximately 1,5 cm in diameter. Histopathological examination revealed HCC in the larger mass lesion with a lower ^18^F-FDG uptake, and cholangiocellular carcinoma in the smaller mass lesion with a higher ^18^F-FDG uptake. To our knowledge, this is the first case report of two histopathologically different primary malignant liver tumors in two distinct segments of the liver detected by PET/CT.

## Abstract (tr)

Radyolojik görüntülemede karaciğer sağ lob segment 5’de hepatosellüler karsinom (HCC) ile uyumlu kitle lezyonu saptanan ve alfa-fetoprotein yüksekliği (323 ng/mL) olan 43 yaşında erkek bir hasta kliniğimize başvurdu. ^18^F-FDG pozitron emisyon tomografi/bilgisayarlı tomografi (PET/BT) görüntüleri, kitle lezyonda fizyolojik seviyede ^18^F-FDG tutulumu gösterdi. Ek olarak, sol lob segment 2’de artmış ^18^F-FDG tutulumu olan, yaklaşık 1,5 cm çapında ikinci bir odak tespit edildi. Histopatolojik incelemede, daha düşük ^18^F-FDG tutan büyük kitle lezyonda HCC, daha yüksek ^18^F-FDG tutan küçük kitle lezyonda ise kolanjiosellüler karsinom saptandı. Bildiğimiz kadarıyla bu, PET/BT ile tanısı konulmuş karaciğerin farklı iki segmentinde histopatolojik olarak farklı iki primer malign karaciğer tümörünün bildirildiği ilk olgu sunumudur.

## Figures and Tables

**Figure 1:**
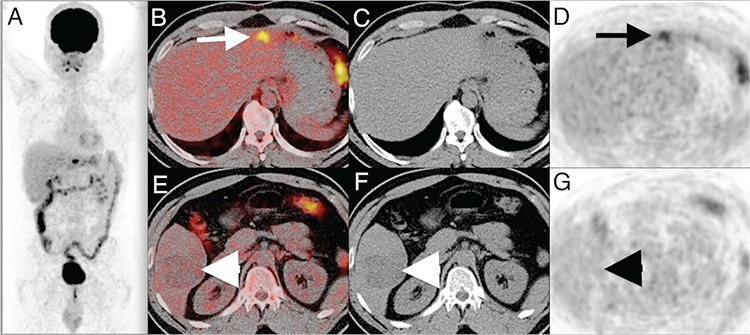
Positron emission tomography/computed tomography (PET/CT) images were acquired after a long period of fasting and 370 MBq (10 mCi) ^18^F-FDG administration. Maximum intensity projection (A) and axial slices of fusion (B, arrow), CT (C) and PET (D) images showed focal hyper-metabolic lesion in the left lobe, segment 2. A mass lesion, approximately 7 cm in diameter, with heterogeneous density at the right liver lobe segment 5 was detected, that displayed normal ^18^F-FDG uptake levels as the liver parenchyma (E arrowhead, F arrowhead, G). PET/CT did not reveal any other focus that was suspicious for malignancy. Hepatocellular carcinoma (HCC) and cholangiocellular carcinoma (CCC) are both primary malignant liver tumors originating from the hepatocyes and bile duct cells, respectively. The incidence of HCC and CCC together is extremely rare (lower than 1% of all primary malignant liver tumors) (1). Synchronous HCC and CCC cases, some of them on the same segment, detected with CT or magnetic resonance imaging (MRI) studies have previously been reported (2,3). Besides, both HCC and CCC components present in the same tumor and hepatic stem cells differentiating to hepatocytes or cholangio cells have also been reported (3). To our knowledge, this is the first case to report synchronous primary malignant liver tumors in two distinct segments detected by ^18^F-FDG PET/CT. Cancer cell growth depends mainly on glucose metabolism. ^18^F-FDG uptake in malignant tumors is related to glucose transporter proteins (especially Glut1) and hexokinase type 2. Glut1 expression is low in HCC and high in CCC, while hexokinase 2 expression is elevated in HCC (4). ^18^F-FDG uptake is variable in HCC related to the degree of differentiation. Because glucose-6-phosphatase activity is high in well differentiated hepatocyte cells, intracellular ^18^F-FDG-6-phosphate is dephosphorylated to ^18^F-FDG, thus decreasing intracellular accumulation (5). Increased ^18^F-FDG uptake were reported in nearly half of the HCC cases. The higher ^18^F-FDG uptake of intrahepatic CCC and lower ^18^F-FDG uptake of hilar tumors is well known. CCC located in the hilum mostly originate from larger bile ducts, so obstructive symptoms are observed in the early periods. The low ^18^F-FDG uptake can thus be attributed to the small tumor size at diagnosis. Other reasons for the low ^18^F-FDG uptake by this tumor have been reported as mucin accumulation inside tumor cells or neoplastic glandular tissue lumen, and scattered settlement of malignant cells in fibrous stroma (6,7). In our case, ^18^F-FDG uptake patterns of both tumors were quite different, so we considered two separate HCC lesions with two distinct degree of differentiation. Histopathologically, the mass lesion with low ^18^F-FDG uptake at the right liver lobe segment 5 was reported as clear cell HCC with micro-macro trabecular pattern, nuclear grade 2-3, while the tumor with higher ^18^F-FDG uptake at the left liver lobe segment 2 was identified as adenocarcinoma (CCC). The CCC was intrahepatic and therefore the ^18^F-FDG uptake was significantly higher.
